# Endocrine Immune-Related Adverse Events Are Independent Predictors of Survival in Patients with Lung Cancer

**DOI:** 10.3390/cancers16091764

**Published:** 2024-05-01

**Authors:** Emmanouil Panagiotou, Sofia Ntouraki, Ioannis A. Vathiotis, Maria Effrosyni Livanou, Athanasios Trimis, Georgios Evangelou, Andriani Charpidou, Konstantinos Syrigos, Melpomeni Peppa

**Affiliations:** 1 Third Department of Medicine, Sotiria General Hospital for Chest Diseases, National and Kapodistrian University of Athens, 11527 Athens, Greece; 2 Endocrine Unit, Second Propaedeutic Department of Internal Medicine, Research Institute and Diabetes Center, Attikon University Hospital, School of Medicine, National and Kapodistrian University of Athens, 11527 Athens, Greece

**Keywords:** CTLA-4, diabetes mellitus, endocrine toxicity, immune checkpoint inhibitors, immune-related adverse events, non-small-cell lung cancer, PD-1, PD-L1, pituitary disorders, small-cell lung cancer, thyroid disorders

## Abstract

**Simple Summary:**

Lung cancer is a global health concern, but survival rates have improved due to novel treatments, including immunotherapy. Treatment with immune checkpoint inhibitors can lead to immune-related adverse events (irAEs), which occur due to inappropriate activation of the immune system. We conducted a retrospective study in patients with lung cancer who received treatment with immune checkpoint inhibitors over a time span of 10 years at a tertiary referral center. Endocrine irAEs (e-irAEs) were common and occurred early in the course of treatment. Importantly, patients with e-irAEs lived longer than patients without; this association was observed across histologic subtypes and was not related to the duration of treatment with immune checkpoint inhibitors.

**Abstract:**

Lung cancer (LC) is a serious health problem worldwide. Survival outcomes have improved over time due to the widespread use of novel therapeutic agents, including immune checkpoint inhibitors (ICIs). Endocrine immune-related adverse events (e-irAEs) are common in LC patients treated with ICIs. We performed a retrospective study of patients with LC who received treatment with ICIs at a tertiary referral center between January 2014 and October 2023. In total, 983 LC patients were included in the study. E-irAEs presented at a median time of 4.1 months and included hypothyroidism (15.6%), hyperthyroidism (4.3%), adrenal insufficiency (0.4%), hypophysitis (0.4%), and diabetes mellitus (0.2%). These toxicities were not related to the duration of treatment or the type of ICIs. Most (97.6%) e-irAEs were mild (grade 1–2). Median overall survival (OS) was higher in LC patients who experienced e-irAEs (31.6 months) compared to those who did not (10.8 months). The difference remained statistically significant in the 3-month (HR: 0.42) and 6-month landmark analysis (HR: 0.51). The OS advantage was observed in both patients with NSCLC (HR: 0.36) and SCLC (HR: 0.27). Additional research is needed to validate the role of e-irAEs as an independent predictor of survival outcomes in patients with LC.

## 1. Introduction

Lung cancer (LC) is the leading cause of cancer-related mortality worldwide, accounting for approximately 1.8 million deaths in 2020 [1]. While the prognosis remains poor in patients with advanced or metastatic disease, survival rates have been increasing over time [2] due to the widespread adoption of novel therapeutic agents, including immune checkpoint inhibitors (ICIs). Monoclonal antibodies targeting programmed cell death protein- or ligand-1 (PD-1/PD-L1) and cytotoxic T-lymphocyte antigen-4 (CTLA-4) have altered the treatment landscape of advanced or metastatic non-small-cell lung cancer (NSCLC) as well as extensive-stage small-cell lung cancer (SCLC) and represent the standard of care for the majority of cases [3]. The antitumor activity of ICIs arises from the suppression of tumor immune evasion and the promotion of T-cell activation [4]. 

Adverse events related to treatment with ICIs are largely the result of aberrant immune activation, causing a different toxicity profile compared to cytotoxic chemotherapy [5,6]. Immune-related adverse events (irAEs) can affect various organ systems, most commonly the skin, gastrointestinal tract, and endocrine glands [5,6]. As a matter of fact, endocrine irAEs (e-irAEs) may affect 10–40% of patients receiving treatment with ICIs [7,8]. The two main endocrinopathies observed are hypophysitis and primary thyroid dysfunction, with hypothyroidism being significantly more frequent than hyperthyroidism. Less common manifestations include autoimmune diabetes mellitus and primary adrenal insufficiency [9]. The incidence of different endocrinopathies varies by ICI class and is significantly higher in patients receiving combination therapy [7]. E-irAEs usually present within 6 months from the initiation of therapy [9,10]. Importantly, the development of e-irAEs has been associated with an improved response to ICI therapy [11,12].

In this retrospective study, we evaluated the incidence of e-irAEs, their association with clinical and pathologic characteristics, and their impact on survival outcomes in a large cohort of patients with LC.

## 2. Materials and Methods

Eligible patients had cytologically or histologically confirmed LC and received at least one treatment cycle (PD-1, PD-L1, or CTLA-4 inhibitor) for locally advanced or metastatic disease between January 2014 and October 2023 at the Third Department of Medicine, “Sotiria” General Hospital for Chest Diseases, Athens, Greece. Data were collected retrospectively. The date of data cutoff was 1 December 2023. Demographic data, disease features, and follow-up information were collected from patient records and hospital registries using a standardized electronic form. 

Disease was staged according to the eighth edition of the American Joint Committee on Cancer (AJCC) staging criteria for LC. The Common Terminology Criteria for Adverse Events (CTCAE), v. 5.0, were used to classify and grade irAEs. The Eastern Cooperative Oncology Group Performance Status (ECOG PS) scale was used to evaluate patient functional status. The objective response rate (ORR) was evaluated using version 1.1 of the Response Evaluation Criteria in Solid Tumors (RECIST, v1.1). Progression-free survival (PFS) was defined as the time from treatment initiation to disease progression according to RECIST, v.1.1, or death from any cause, whichever occurred first. Overall survival (OS) was defined as the time from treatment initiation to death from any cause. Information about subsequent treatments was also collected. 

## 3. Statistical Analysis

Descriptive statistics were utilized to analyze patient demographics and disease characteristics. PFS and OS were calculated with Kaplan–Meier analysis. The median time of follow-up was calculated by reversing events and censored data. Associations between patient characteristics and outcomes were analyzed using Cox proportional hazards regression analysis. Three-month and six-month landmark analysis was performed in an effort to minimize immortal time bias. Binomial logistic regression analysis was performed to identify associations of different variables with e-irAEs. Missing values were excluded for the Cox proportional hazards and binomial logistic regression analyses. Variables with a *p*-value < 0.1 in the univariate analysis were included in the multivariate analyses. Hypothesis testing was conducted at a two-sided significance level of α = 0.05. All analyses were performed in R (version 4.2.1).

## 4. Results

A total of 983 LC patients were included in this study. The median age of study participants was 67 years (range, 22–93). Of those patients, 738 patients (75.1%) were males and 245 patients (24.9%) were females. Regarding smoking status, 595 patients (67.1%) were current smokers, while 265 patients (29.9%) and 27 patients (3%) were former and never smokers, respectively (Table 1).

At baseline, 696 patients (73.2%) had an ECOG PS score of 0 or 1, followed by 211 patients (22.2%) with an ECOG PS score of 2 and 44 patients (4.6%) with a score of 3 or more. As far as histologic subtype is concerned, 474 patients (48.3%) had adenocarcinoma, 280 patients (28.5%) had squamous cell carcinoma, 142 patients (14.5%) had SCLC, 31 patients (3.2%) had lung carcinoma not otherwise specified (NOS), 17 patients (1.7%) had large-cell neuroendocrine carcinoma (LCNEC), and 37 patients (3.8%) had other lung carcinomas.

At the time of treatment with ICIs, the stage distribution was as follows: Stage II, 2 patients (0.2%); Stage IIIA, 36 patients (3.7%); Stage IIIB, 62 patients (6.3%); Stage IIIC, 17 patients (1.7%); Stage IVA, 357 patients (36.4%); and Stage IVB, 508 patients (51.7%). Metastatic lesions were present in the contralateral lung in 383 patients (39%), pleura in 348 patients (35.4%), brain in 188 patients (19.1%), bones in 310 patients (31.5%), liver in 194 patients (19.7%), adrenal glands in 182 patients (18.5%), and other sites in 309 patients (31.5%).

Most patients (56.4%) received treatment with ICIs as first-line therapy, followed by second-line (27.9%), third-line, or later-line (9.4%), and consolidation therapy after concurrent chemoradiotherapy (6.3%). The PD-L1 tumor proportion score (TPS) was negative (<1%) in 27.1%, low (1–49%) in 37.2%, and high (≥50%) in 35.7% of evaluated patients. Regarding the type of immunotherapy, 670 patients (68.1%) received treatment with a PD-1 inhibitor, 221 patients (22.5%) with a PD-L1 inhibitor, and 92 patients (9.4%) combined checkpoint blockade with a PD-1 and a CTLA-4 inhibitor. Also, approximately half (49.2%) of the patients received chemotherapy concurrently with immunotherapy (Table 2).

At the time of data cutoff, the median follow-up was 22.6 months (95% CI, 19.9–25.2 months). Tumor response was evaluable in 902 patients, while 569 patients (58.0%) had died. The ORR was 22.8%, while the clinical benefit rate (CBR) was 50.3%, with six patients (0.7%) achieving a complete response and two-hundred patients (22.1%) achieving a partial response. At the time of data cutoff, 737 patients (75.8%) had experienced disease progression or death. The median PFS was 5.17 months (95% CI, 4.4–5.6 months). The median OS was 12.8 months (95% CI, 11.7–14.4 months).

In multivariate analysis, age lower than 65 years, PD-L1 expression, concurrent radiotherapy, endocrine/non-endocrine irAEs, and white blood cell count were associated with higher OS; while performance status, pulmonary comorbidities, pleural/adrenal metastases, neutrophil/lymphocyte count and high-serum LDH were associated with lower OS (Table 3).

### 4.1. irAEs

A total of 513 patients (59.6%) experienced at least one irAE. The most common type of irAE was dermatologic (25.6%), followed by endocrine (20.9%), gastrointestinal (17.6%), respiratory (11.0%), hepatic (5.7%), and other (27.9%). All six patients (100%) that demonstrated a complete response (CR) to treatment experienced at least one irAE.

### 4.2. E-irAEs

**Figure 1:**
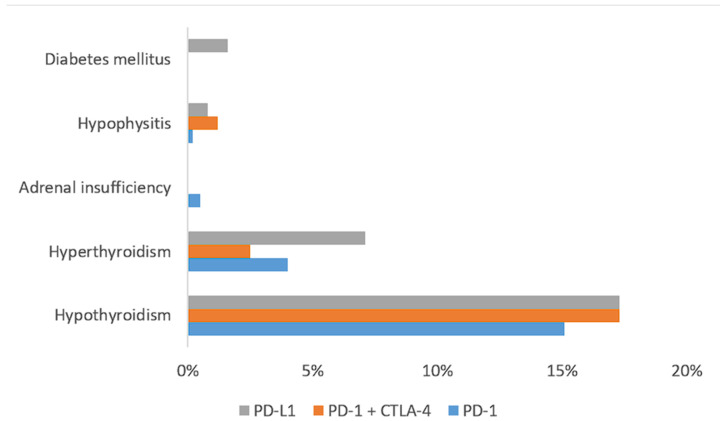
Incidence of endocrine immune-related adverse events, depending on the class of immune checkpoint inhibitor.

E-irAEs were distributed as follows: hypothyroidism, one-hundred and thirty patients (15.6%); hyperthyroidism, thirty-six patients (4.3%); adrenal insufficiency, three patients (0.4%); hypophysitis, three patients (0.4%); and diabetes mellitus, two patients (0.2%). The incidence of endocrine irAEs, depending on the type of checkpoint inhibitor received, is summarized in Figure 1.

**Figure 2:**
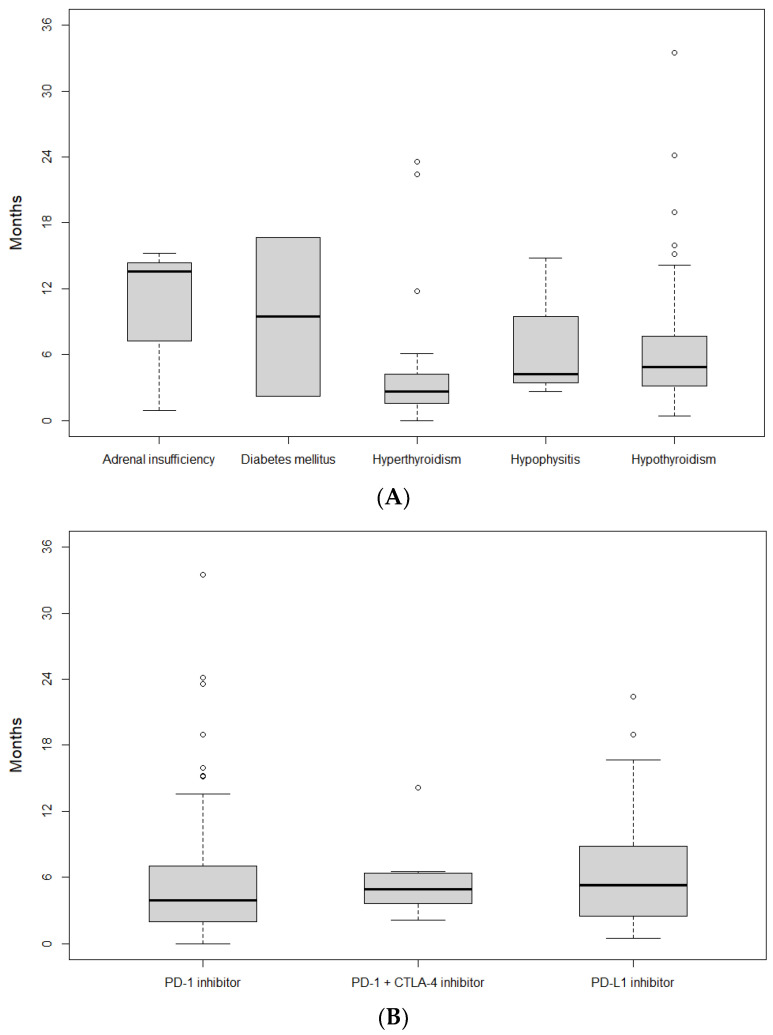
Onset of endocrine immune-related adverse events (irAEs), depending on the type of irAEs (**A**) and the class of immune checkpoint inhibitors (**B**).

Most e-irAEs were mild (grade 1–2, 97.6%), with four patients (2.4%) experiencing a grade 3 event (hypothyroidism, adrenal insufficiency, hypophysitis, and diabetes mellitus; one patient each). The development of e-irAEs led to temporary treatment discontinuation in thirteen patients (7.6%) and permanent treatment discontinuation in one patient (0.6%), who had grade 2 hyperthyroidism. The median time from treatment initiation until the development of e-irAEs was 4.1 months. The median time-to-onset of thyroid disease showed a statistically significant difference (*p* = 0.004) between hypothyroidism (4.87 months; 95% CI, 4.07–5.63 months) and hyperthyroidism (2.62 months; 95% CI, 2.10–3.77 months). The onset of e-irAEs depending on the type and ICI class is summarized in Figure 2A and Figure 2B, respectively.

Patients who demonstrated an objective response to treatment were significantly more likely to experience an e-irAE (35.0% vs. 18.1%; OR, 2.43; 95% CI, 1.65–3.55; *p* = 3.59 × 10^−6^). However, the distribution of e-irAEs was similar between responders and non-responders (*p* = 0.3). Univariate analysis showed a trend towards increased risk of e-irAEs for women (OR, 1.39; 95% CI, 0.95–2.02; *p* = 0.09), although it was not statistically significant in multivariate analysis (*p* = 0.8). In multivariate analysis, the presence of e-irAEs was associated with the presence of endocrine comorbidities at diagnosis, the absence of liver metastases, and objective response to treatment (Table 4).

**Figure 3:**
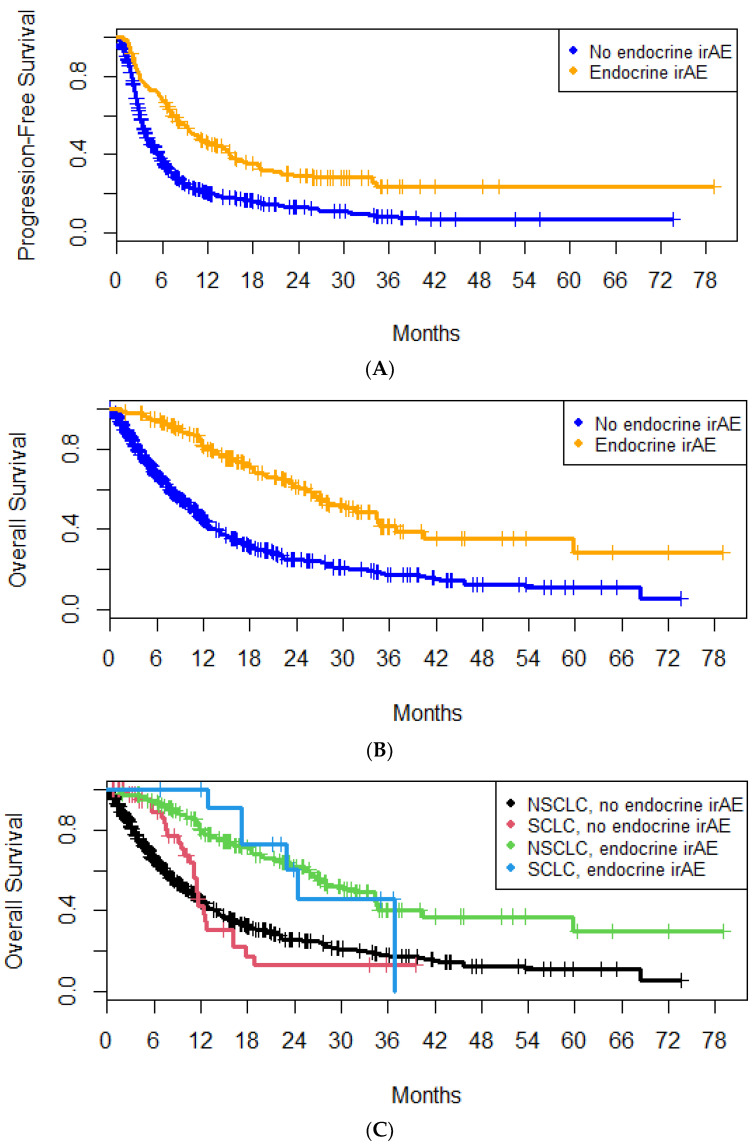
Overall survival of patients with or without endocrine immune-related adverse events in the entire cohort (**A**) and depending on the tumor type (**B**); progression-free survival in patients with or without endocrine immune-related adverse events (**C**).

Median PFS was increased in patients who experienced e-irAEs (10.7 months; 95% CI, 8.4–14.7 months) compared to patients who did not (3.8 months; 95% CI, 3.4–4.4 months) (Figure 3A). The hazard ratio (HR) for disease progression or death was 0.50 (95% CI, 0.41–0.61; *p* = 10^−11^). Median PFS was increased in NSCLC patients with e-irAEs (10.7 vs. 3.6 months; HR, 0.48; 95% CI, 0.39–0.59; *p* = 1.55 × 10^−11^). However, this was not the case for patients with SCLC (7.3 vs. 7.0 months; HR, 0.81; 95% CI, 0.40–1.66; *p* = 0.57).

Median OS was increased in patients who experienced e-irAEs (31.6 months; 95% CI, 25.9–40.5 months) compared to patients who did not (10.8 months; 95% CI, 9.47–11.7 months) (Figure 3B). The HR for death was 0.36 (95% CI, 0.28–0.46; *p* = 1.56 × 10^−15^). The survival difference between the two subgroups remained statistically significant in the 3-month (HR, 0.42; 95% CI, 0.33–0.55; *p* = 2 × 10^−11^) and 6-month landmarks (HR, 0.51; 95% CI, 0.39–0.66; *p* = 3 × 10^−7^). Importantly, the OS advantage was observed in patients with both NSCLC (31.6 vs. 10.4 months; HR, 0.36; 95% CI, 0.28–0.47; *p* = 3.14 × 10^−14^) and SCLC (24.4 vs. 11.6 months; HR, 0.27; 95% CI, 0.11–0.66; *p* = 0.004) (Figure 3C).

In the multivariate analysis, age < 65 years, PD-L1 expression, concurrent radiotherapy, endocrine/non-endocrine irAEs, and white blood cell count were associated with higher OS; while performance status, pulmonary comorbidities, pleural/adrenal metastases, neutrophil/lymphocyte count, and high serum LDH were associated with lower OS (Table 4).

### 4.3. Non-Endocrine irAEs

**Figure 4:**
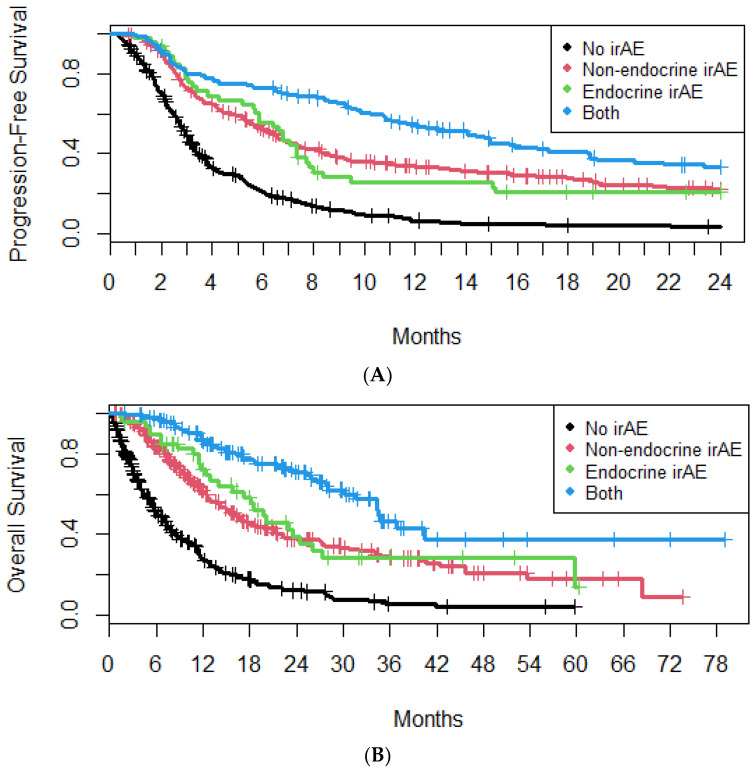
Progression-free survival (**A**) and overall survival (**B**) of patients with or without endocrine or non-endocrine immune-related adverse events.

Median PFS was increased in patients with both endocrine and non-endocrine irAEs (14.1 months; 95% CI, 10.8–18.9 months), compared to patients with only endocrine (6.8 months; 95% CI, 5.6–8.0 months) or non-endocrine irAEs (6.4 months; 95% CI, 5.6–7.4 months) (Figure 4A). Median OS was increased in patients with both endocrine and non-endocrine irAEs (34.5 months; 95% CI, 30.2 months–NA), compared to patients with only endocrine (19.8 months; 95% CI, 15.9–27.3 months) or non-endocrine irAEs (16.1 months; 95% CI, 14.0–21.0 months) (Figure 4B). 

### 4.4. Corticosteroids

A total of 27 patients (15.7%) received corticosteroids for the treatment of e-irAEs. The indication for corticosteroid therapy was distributed as follows: hypothyroidism, ten patients (7.8%); hyperthyroidism, fourteen patients (38.9%); hypophysitis, two patients (66.7%); adrenal insufficiency, one patient (33.3%). Median OS was decreased in patients who received corticosteroids for the treatment of e-irAEs (23.0 vs. 34.4 months; HR, 1.90; 95% CI, 1.11–3.25; *p* = 0.019), while median PFS was similar between the two subgroups (11.7 vs. 10.2 months; HR, 1.06; 95% CI, 0.64–1.75; *p* = 0.8).

## 5. Discussion

This retrospective study demonstrated the presence of e-irAEs in 20.9% of patients with LC receiving treatment with ICIs. Importantly, e-irAEs were associated with prolonged PFS and OS irrespective of the histologic subtype or established prognostic factors. 

Incidence rates of e-irAEs have been previously reported in the literature [9]; no new safety signals were demonstrated in this study. The incidence rate of different e-irAEs is dependent on the class of ICI [13]. In particular, hypophysitis is more common in patients treated with CTLA-4 inhibitors, while thyroid, adrenal, and pancreas-related side effects are more common with PD-1/PD-L1 inhibitors; the incidence of e-irAEs increases with the combination of PD-1 and CTLA-4 inhibitors [14]. In our study, no significant differences in the incidence of e-irAEs were observed between PD-1 inhibitors, PD-L1 inhibitors, and the combination of PD-1 and CTLA-4 inhibitors, which might be attributed to the small number of patients in the subgroups excluding thyroid e-irAEs. In accordance with the literature, multivariate analysis revealed that prior history of endocrine disease was associated with the development of e-irAEs. Pre-existing endocrinopathy has been previously shown to increase the risk of endocrine irAEs [15], and this particularly relates to thyroid dysfunction in patients with autoimmune thyroid disease [16].

Although younger age and female sex have been suggested to increase the risk of e-irAEs [17], a trend towards higher risk in women was identified in the univariate analysis but did not reach statistical significance in the multivariate setting. Immune-related hyperthyroidism occurred on average two months earlier than hypothyroidism in our study, which is also consistent with previous studies [12]. The timeline of thyroid toxicities could be interpreted by the fact that thyrotoxicosis is, in many cases, the initial presentation of subsequent hypothyroidism [14]. The underreporting of thyrotoxicosis relative to hypothyroidism might be attributed to the typically mild or asymptomatic nature of the transient thyrotoxic phase, which is often not recorded in real-world studies [18,19].

In general, e-irAEs do not require the discontinuation of ICIs and rarely require corticosteroid therapy. However, unlike other irAEs, they tend to be irreversible, leading to a need for lifelong hormone supplementation [14,18,20]. The impact of corticosteroid therapy for irAEs on ICI efficacy is unclear; the organ system affected, the primary tumor type, as well as the timing and dose of systemic steroid therapy may be significant [21,22,23,24]. It has been suggested that the use of systemic corticosteroids is a poor prognostic factor independent of the use of ICIs; however, this has not been consistently shown in patients with LC [25,26]. To our knowledge, the adverse association between corticosteroid use for endocrine irAEs and OS shown in our study has not been demonstrated before. While limited conclusions can be drawn, given the small number of patients, this finding may warrant further study.

Targeting tumor immune evasion, a recognized hallmark of carcinogenesis, has been consistently shown to be an effective strategy for improving survival outcomes in patients with cancer [27]. The development of irAEs has been associated with ICI efficacy in various tumor types, including LC [28]. In our study, the development of e-irAEs was associated with improved PFS and OS in patients with LC, and the association was statistically significant in both the multivariate and landmark analyses. E-irAEs have been consistently correlated with improved survival outcomes in several real-world observational studies [12]. Two recent meta-analyses of studies in patients with LC receiving ICIs have demonstrated different effect sizes for the impact of e-irAEs on OS. In the study by Cheung et al., the effect size was similar to that in our study (HR, 0.37; 95% CI, 0.24–0.57), while in the meta-analysis by Wang et al., the beneficial effect of e-irAEs was smaller (HR, 0.55; 95% CI, 0.45–0.67) [12,29]. This discrepancy may be partially attributed to different patient populations, as almost half (49.2%) of the patients in our study also received chemotherapy in combination with ICIs. Furthermore, a significant proportion of SCLC patients were included, a population which was underrepresented in previous analyses and derived a clinically meaningful survival advantage from the presence of e-irAEs. Interestingly, the impact of e-irAEs on survival was independent of the development of non-endocrine irAEs, potentially suggesting additive biological mechanisms.

Shared mechanisms between the development of irAEs and the mechanism of action of ICIs have been suggested as a biological explanation for the observed association between the presence of irAEs and ICI efficacy [28,30]. The precise pathophysiology behind the development of irAEs has not been identified. Potential mechanisms include enhanced T-cell activity and the presence of pro-inflammatory cytokines or autoantibodies [31]. Genetic susceptibility and the gut microbiome may also be involved [32]. Different underlying mechanisms may explain the difference in the incidence of irAEs by the class of ICI regimens [31,32].

Certain factors may complicate the use of e-irAEs as a surrogate endpoint for ICI efficacy. Immortal time bias affects survival analyses stratified by the presence of treatment-related toxicity [33]. This may be particularly prominent in cases of delayed-onset toxicity or diseases with poor survival. However, the association of e-irAEs with prolonged PFS and OS remained statistically significant after controlling for immortal time bias by conducting a landmark analysis. In addition, e-irAEs may occur several months after treatment initiation [34], as in our study, allowing for a radiological assessment of tumor response. Still, the utility of response rate has been questioned in ICI studies [35]; the rate of irAEs may prove useful as an additional marker of response. A meta-analysis of randomized clinical trials has questioned the role of irAEs as a surrogate for ICI efficacy in solid tumors. However, an association between mild (grade 1–2) irAEs and OS was observed among patients with NSCLC with PD-L1 TPS ≥ 1% [36]. 

To our knowledge, this is the largest retrospective study of e-irAEs in patients with LC. However, this study has several limitations. The retrospective, single-center design limits inferences about causality as well as the generalizability of the study results. In addition, although the number of included patients was sufficient, we did not observe any association between ICI class and select rare e-irAEs, including hypophysitis, adrenal insufficiency, and diabetes mellitus; it should be noted that the absence of an association between ICI class and thyroid irAEs could not be attributed to the sample size [13]. Furthermore, as the data were collected retrospectively from patient records, missing or incomplete records may bias the results.

## 6. Conclusions

The incidence and characteristics of e-irAEs in LC patients treated with ICIs were generally consistent with those in the published literature. Preexisting endocrine disease and the absence of liver metastases were associated with incidence. The development of e-irAEs was associated with increased PFS and OS. The OS benefit remained statistically significant in multivariate and landmark analyses. Prospective studies are needed to validate the association between e-irAEs and survival outcomes as well as incorporate e-irAEs in ICI response assessment.

## Figures and Tables

**Table 1:** Patient demographics.

	Subgroup	N (%)
Sex		
	Female	245 (24.9%)
	Male	738 (75.1%)
Age	≥65	570 (58%)
	<65	413 (42%)
Smoking status		
	Current	595 (67.1%)
	Former	265 (29.9%)
	Never	27 (3%)
Comorbidity		
	Cardiac	642 (67.9%)
	Endocrine	322 (34%)
	Pulmonary	255 (27%)
	Gastrointestinal	88 (9.4%)
	Renal	53 (5.6%)
	Rheumatologic	20 (2.1%)
ECOG PS		
	0	198 (20.8%)
	1	498 (52.4%)
	2	211 (22.2%)
	≥3	44 (4.6%)
Prior treatments		
	Surgery	166 (17%)
	Radiotherapy	427 (44.3%)
	Chemotherapy	472 (48.6%)
Treatment line		
	Consolidation	61 (6.3%)
	1	549 (56.4%)
	2	272 (27.9%)
	≥3	92 (9.4%)

Abbreviations: ECOG PS, Eastern Cooperative Oncology Group Performance Status.

**Table 2:** Disease characteristics.

	Subgroup	N (%)
Tumor specimen		
	Tissue	912 (92.9%)
	Cytology	70 (7.1%)
Histologic subtype		
	Adenocarcinoma	474 (48.3%)
	Squamous	280 (28.5%)
	SCLC	142 (14.5%)
	NSCLC NOS	31 (3.2%)
	LCNEC	17 (1.7%)
	Other	37 (3.8%)
PD-L1		
	<1%	180 (27.1%)
	1–49%	247 (37.2%)
	≥50%	237 (35.7%)
Driver mutations		
	No	368 (71.8%)
	Yes	144 (28.2%)
Stage at treatment initiation		
	II	2 (0.2%)
	IIIA	36 (3.7%)
	IIIB	62 (6.3%)
	IIIC	17 (1.7%)
	IVA	357 (36.4%)
	IVB	508 (51.7%)
Metastatic sites		
	Contralateral Lung	383 (39%)
	Pleura	348 (35.4%)
	Brain	188 (19.1%)
	Bone	310 (31.5%)
	Liver	194 (19.7%)
	Adrenal gland	182 (18.5%)
	Other	309 (31.5%)
Checkpoint inhibitor		
	PD-1 inhibitor	670 (68.1%)
	PD-L1 inhibitor	221 (22.5%)
	PD-1 + CTLA-4 inhibitor	92 (9.4%)
Concurrent chemotherapy		
	No	499 (50.8%)
	Platinum-pemetrexed	211 (21.5%)
	Platinum-etoposide	157 (16%)
	Platinum-taxane	103 (10.5%)
	Single-agent chemotherapy	12 (1.2%)
Concurrent radiotherapy		193 (19.8%)
Objective Response		
	Complete response (CR)	6 (0.7%)
	Partial response (PR)	200 (22.1%)
	Stable disease (SD)	249 (27.5%)
	Progressive disease (PD)	450 (49.7%)
Subsequent therapy		
	Chemotherapy	345 (89.8%)
	Targeted therapy	17 (4.4%)
	Chemoimmunotherapy	14 (3.7%)
	Radiotherapy	5 (1.3%)
	Immunotherapy	3 (0.8%)

Abbreviations: LCNEC, large-cell neuroendocrine carcinoma; NSCLC NOS, non-small-cell lung cancer not otherwise specified; SCLC, small-cell lung cancer.

**Table 3:** Univariate and multivariate Cox proportional hazards analysis, evaluating associations between different variables and overall survival.

		Univariate Analysis	Multivariate Analysis	
	Subgroup	*p*-Value	*p*-Value	HR (95% CI)
Age	<65	0.021	0.0012	0.54 (0.37–0.78)
BMI	Normal	(ref)		
	Overweight	0.023		
	Obese			
ECOG PS	0	(ref)		
	1	8.07 × 10^−8^		
	2	1.18 × 10^−22^	0.016	2.46 (1.18–5.09)
	3	3.24 × 10^−16^	0.014	3.43 (1.28–9.18)
	4	0.0007		
Pulmonary comorbidity		0.063	0.042	1.52 (1.02–2.27)
Endocrine comorbidity		0.094		
Histologic subtype	Adenocarcinoma	(ref)		
	Squamous	0.00019		
	SCLC			
	Pleomorphic			
	NOS			
	LCNEC			
Stage at diagnosis	I	(ref)		
	II			
	IIIA	0.056		
	IIIB			
	IIIC	0.037		
	IVA			
	IVB	0.029		
Metastases	Lung	0.002		
	Pleura	4.67 × 10^−8^	0.010	1.64 (1.13–2.38)
	Liver	1.07 × 10^−10^		
	Brain	8.86 × 10^−5^	0.067	1.61 (0.97–2.67)
	Bone	2.20 × 10^−7^		
	Adrenal gland	0.027	0.012	1.88 (1.15–3.07)
	Other	0.029		
PD-L1	<1%	(ref)		
	1–49%	0.011		
	≥50%	0.040	0.001	0.44 (0.27–0.71)
Prior surgery		0.0002		
Prior radiotherapy		0.024		
Prior chemotherapy		4.33 × 10^−7^		
Treatment line	1	(ref)		
	2	2.72 × 10^−10^		
	3	1.70 × 10^−9^		
	4	4.12 × 10^−5^		
	5			
	Consolidation	0.0039		
Checkpoint inhibitor	PD-1 inhibitor	(ref)		
	PD-L1 inhibitor	5.97 × 10^−4^		
	PD-1 + CTLA-4 inhibitor	0.025	0.053	0.49 (0.24–1.01)
Concurrent radiotherapy		0.0012	0.018	0.57 (0.36–0.91)
Endocrine irAE		1.56 × 10^−15^	0.005	0.48 (0.28–0.80)
Non-endocrine irAE		2.92 × 10^−28^	2.88 × 10^−8^	0.34 (0.23–0.50)
White blood cell count		7.72 × 10^−6^	0.0008	0.9993 (0.99889–0.9997)
Platelets		0.033		
Neutrophils		2.21 × 10^−8^	0.0006	1.001 (1.00032–1.0012)
Lymphocytes		0.087	0.009	1.001 (1.00018–1.0013)
NLR	<2	(ref)		
	2–3			
	>3	9.94 × 10^−9^		
LDH	>ULN	1.96 × 10^−6^	0.034	1.6 (1.04–2.48)
Hemoglobin	≥12 mg/dL	1.94 × 10^−6^		

Results reported for associations with *p* < 0.1. Abbreviations: BMI, body mass index; ECOG PS, Eastern Cooperative Oncology Group Performance Status; irAE, immune-related adverse event; LCNEC, large -ell neuroendocrine carcinoma; LDH, lactate dehydrogenase; NLR, neutrophil-to-lymphocyte ratio; NOS, not otherwise specified; SCLC, small-cell lung cancer; ULN, upper limits of normal.

**Table 4:** Univariate and multivariate binomial logistic regression analysis, evaluating associations between different variables and endocrine immune-related adverse events.

		Univariate Analysis	Multivariate Analysis	
	Subgroup	*p*-Value	*p*-Value	OR (95% CI)
Sex	Male	0.091		
ECOG PS	0	(ref)		
	1			
	2	0.0014	0.0955	0.42 (0.15–1.16)
	3	0.0648		
	4			
Endocrine comorbidity		0.0168	0.0061	2.10 (1.24–3.58)
Histology	Adenocarcinoma	(ref)		
	Adenosquamous	0.0144		
	SCLC			
	LCNEC	0.0948		
	NOS			
	Pleomorphic	0.0145		
	Squamous			
Bone metastases		0.0998		
Liver metastases		0.0002	0.0383	0.33 (0.11–0.94)
PD-L1 TPS	<1%	(ref)		
	1–49%	0.074		
	≥50%			
Prior surgery		0.004	0.0619	1.80 (0.97–3.35)
Treatment line	1	(ref)		
	2			
	3			
	4			
	5			
	Consolidation	0.0978		
Checkpoint inhibitor	PD-1 inhibitor	(ref)		
	PD-L1 inhibitor			
	PD-1 + CTLA-4 inhibitor	0.0702		
Concurrent radiotherapy		0.0741		
Concurrent chemotherapy	No	(ref)		
	Platinum-etoposide			
	Platinum-pemetrexed			
	Platinum-taxane	0.0381		
	Single-agent			
Objective response	SD/PD	(ref)		
	CR/PR	3.59 × 10^−6^	0.0455	1.84 (1.01–3.34)
Dermatologic irAE		3.03 × 10^−8^	0.051	1.76 (1.00–3.10)
Pulmonary irAE		0.045		
Liver irAE		0.0049		
Other irAE		4.13 × 10^−7^		
Neutrophils		0.0527		
NLR	<2	(ref)		
	2–3			
	>3	0.009		

Results reported for associations with *p* < 0.1. Abbreviations: ECOG PS, Eastern Cooperative Oncology Group Performance Status; irAEs, immune-related adverse events; LCNEC, large-cell neuroendocrine carcinoma; NLR, neutrophil-to-lymphocyte ratio; NOS, not otherwise specified; SCLC, small-cell lung cancer.

## Data Availability

The data presented in this study are available on request from the corresponding author due to privacy restrictions.
